# *Clostridium*, *Bacteroides* and *Prevotella* associates with increased fecal metabolites *Trans*-4-Hydroxy-L-proline and Genistein in active pulmonary tuberculosis patients during anti-tuberculosis chemotherapy with isoniazid-rifampin-pyrazinamide-ethambutol (HRZE)

**DOI:** 10.1007/s12088-022-01003-2

**Published:** 2022-03-24

**Authors:** Ruijie Meng, Wenya Dong, Jie Gao, Chunrong Lu, Chenchen Zhang, Qinghua Liao, Liang Chen, Huizhong Wu, Jiwen Hu, Wenjing Wei, Zhenyou Jiang

**Affiliations:** 1 Department of Microbiology and Immunology, College of Basic Medicine and Public Hygiene, Jinan University, GuangZhou, 510632 China; 2 Center for Tuberculosis Control of Guangdong Province, Key laboratory of translational medicine of Guangdong, Guangzhou, 510630 China; 3 Shenzhen Center for Chronic Disease Control, Shenzhen, 518102 China; 4 Department of Clinical Laboratory, Guangdong Women and Children Hospital, Guangzhou, 511443 China; 5 Medical Laboratory of Shenzhen Luohu Hospital Group, Shenzhen, 518112 China

**Keywords:** Tuberculosis, Gut microbiome, Anti-TB chemotherapy, 16S rRNA, Fecal metabolome

## Abstract

**Purpose:**

To investigated the changes of gut microbiome and fecal metabolome during anti-tuberculosis chemotherapy with isoniazid (H)-rifampin (R)-pyrazinamide (Z)-ethambutol (E).

**Patients and methods:**

(1) In this study, we recruited 168 stool specimens from 49 healthy volunteers without *M. tuberculosis* (Mtb), 30 healthy volunteers with latently infected by Mtb, 41 patients with active tuberculosis (ATB), 28 patients with 2-month HRZE treatment and 20 patients with 2-month HRZE followed by 4-month HR treatment. (2) We used 16S rRNA sequencing and an untargeted Liquid Chromatograph Mass Spectrometer-based metabolomics to investigate the changes of gut microbiome and the alteration of fecal metabolome, respectively, during anti-TB chemotherapy.

**Results:**

Mtb infection can reduce the diversity of intestinal flora of ATB patients and change their taxonomic composition, while the diversity of intestinal flora of ATB patients were restored during anti-TB chemotherapy. Especially, family *Veillonellacea* and *Bateroidaceae* and their genera *Veillonella* and *Bacteroides* significantly increased in the gut microbiota during anti-TB chemotherapy. Additionally, Mtb infection dynamically regulates fecal metabolism in ATB patients during anti-TB chemotherapy. Interestingly, the altered abundance of fecal metabolites correlated with the altered gut microbiota, especially the change of gut *Clostridium*, *Bacteroides* and *Prevotella* was closely related to the change of fecal metabolites such as *Trans*-4-Hydroxy-L-proline and Genistein caused by Mtb infection or anti-TB chemotherapy.

**Conclusion:**

Anti-TB chemotherapy with HRZE can disrupt both gut microbiotas and metabolome in ATB patients. Some specific genera and metabolites are depleted or enriched during anti-TB chemotherapy. Therefore, revealing potential relevance between gut microbiota and anti-TB chemotherapy will provide potential biomarkers for evaluating the therapeutic efficacy in ATB patients.

**Supplementary Information:**

The online version contains supplementary material available at 10.1007/s12088-022-01003-2.

## Introduction

Tuberculosis (TB) remains a significant human health issue, especially for developing countries. One-third of the world’s population has been infected with *M. tuberculosis* (Mtb)*,* and new infections occur in about 1% of the population each year, of which about 5–10% can further develop into active tuberculosis [1]. It is widely believed that the outcomes of Mtb infection are largely determined by host and environmental factors [2, 3]. Recently, it is supposed that TB may be mainly caused by complex microbial community interactions, not only by Mtb [4]. Furthermore, Mtb infection requires long-term combined antibiotic therapy, which may alter the composition and structure of microbiota, subsequently affecting the outcome of treatment [5]. Thus, studying the changes of human microbiota associated with Mtb infection and anti-TB therapy might play a vital role in understanding the progress and persistence of this disease.

Some recent studies point out that the dysbiosis of gut microbiota may contribute to the lung diseases-related pathophysiological processes, such as the susceptibility, progression and chronicity of TB [6]. A study indicates that aerosol Mtb infection can significantly change gut microbiota in mice [7]. Russell et al*.* also found the streptomycin-exacerbated hypersensitivity pneumonitis was caused by the expansion of streptomycin-resistant Bacteroidetes in the gut microbiota [8]. Another study showed the gut microbiota was a protective mediator of pneumococcal pneumonia by enhancing the function of primary alveolar macrophage [9]. It’s well known that effective treatment of drug-susceptible TB requires at least 6 months of daily administration with a combination of four drugs, namely, rifampin (R), isoniazid (H), pyrazinamide (Z), and ethambutol (E) for 2 months, followed by isoniazid (H) and rifampin (R) for at least 4 months (2HRZE/4HR). Among these drugs, isoniazid, pyrazinamide and ethambutol are specific to the *Mycobacterium* species while rifampin that inhibits RNA polymerase is a broad-spectrum antibiotic against many bacteria. The impact of anti-TB therapy on gut microbiota has recently been explored [5, 10–12]. For example, a recent study in mice has shown that a global change of gut microbiota with HRZE treatment [5]. Furthermore, these alterations resulted from the synergistic action of multiple drugs and RIF has the most significant influence on the outcome of treatment. Accordingly, another three clinical studies also found conventional anti-TB chemotherapy could result in a high level of dysbiosis in the intestinal microbiota [10, 12–14].

To date, few researchers have simultaneously explored the changes of gut microbiome and fecal metabolome in patients with active pulmonary tuberculosis during anti-TB chemotherapy. There are only a few reports on the relationship between plasma metabolites and TB. Researchers found that some metabolites are potentially useful for rapid and noninvasive diagnosis of TB. In 2018, January Weiner 3rd et al*.* revealed that six metabolites (cortisol, glutamine, cotinine, kynurenine, histidine and mannose) in the blood could even be used to predict the onset of tuberculosis [15]. In addition, according to a recent study, four metabolites can be used in combination as potential biomarkers for cured TB [16]. SCFAs strongly modulate immune and inflammatory responses. It has been confirmed that the gut microbiome, which produces short-chain fatty acid (SCFA) metabolites such as butyrate, may affect the host response against Mtb [17]. Feces reflect the final result of the interaction between the entire intestine and the host, so we think that the gut microbiota and metabolites should be taken into consideration together in the diagnostics, treatment and future prevention of TB.

In this study, we present an integrated analysis on TB-associated human gut microbiota and metabolites. Using 16S rRNA sequencing, we firstly investigated the changes of gut microbiome in TB patients during anti-TB chemotherapy with different time points, and found that Mtb infection reduces the diversity and changes the taxonomic compositions of gut microbiota in ATB patients, and the reduced gut microbiota diversity is restored in ATB patients during anti-TB chemotherapy. Moreover, we found that Mtb infection dynamically regulates fecal metabolism in ATB patients during anti-TB chemotherapy, especially the altered abundance of fecal metabolites such as *Trans*-4-Hydroxy-L-proline and Genistein was closely related to the altered gut microbiota *Clostridium*, *Bacteroides* and *Prevotella.*

## Methods and Materials

### Subjects in this Study

We recruited five groups of individuals using a cross-sectional research study design. Characteristics of the study cases are given in Table 1. The detailed screening criteria were shown in Supplemental Experimental Procedures.

**Table 1:** **Characteristics of the participants in this study.** Data are divided into the study groups described in the text. The number of subjects, average age, gender distribution and time on HRZE treatment are shown. Healthy Volunteers are IGRA − , volunteers with LTBI are IGRA + . Active tuberculosis patients were finally diagnosed by positive test of Mtb in sputum specimens

Group^a^	No. of subjects	Age, mean ± SD, year	Male, n (%)	Anti-TB duration	No. of reads	No. of OTUs	Diagnosis
HV	49	40.1 ± 12.4	13 (26.5)	N/A	110,141.3 ± 17,359.3	104.2 ± 27.4	Mtb-/IGRA-
LTBI	30	44.7 ± 14.52	14 (46.7)	N/A	121,480.5 ± 16,342.5	107.8 ± 25.7	Mtb-/IGRA +
ATB	41	35.02 ± 11.83	28 (68.3)	N/A	118,944.5 ± 39,512.1	81.5 ± 33.8	Mtb +
T2	28	34.2 ± 10.78	22 (75)	2 months	108,212.1 ± 14,328.4	69.9 ± 29.1	–
T6	20	38.0 ± 13.5	12 (60)	6 months	95,506.2 ± 19,870.0 1	66.8 ± 22.2	–

^a^HV: Mtb uninfected; LTBI: individuals with latent TB infection; ATB: Active TB; T2: TB patients with anti-TB therapy (HRZE) for 2 months; T6: TB patients with anti-TB therapy (HRZE) for 6 months

### DNA isolation, 16S rRNA Genes Sequencing, and Bioinformatics Analysis

Fecal genomic DNA extraction, 16S rRNA sequencing and bioinformatics analysis were carried out according to conventional methods with some minor modifications. The detailed methods were shown Supplemental Experimental Procedures.

### Metabolite Extraction for LC–MS

Fecal samples were reconstituted with PBS and centrifuged to obtain the supernatant. Supernatant was reconstituted by dissolving in 1 mL solvent mixture containing methanol/acetonitrile (1:1). The samples were further treated and transferred to LC vial for LC–MS analysis. Quality control (QC) samples were prepared and injected at regular intervals (every 8 samples) throughout the analytical run to provide a set of data from which repeatability could be assessed. Please see Supplemental Experimental Procedures for more detailed metods.

### Identification of Metabolites by LC–MS

The separation was performed by Ekspert UltraLC (110, AB Sciex) and equipped with ACQUITY UPLC HSS T3 (1.8 μm 2.1 × 100 mm, Waters) column at a flow rate of 0.3 mL/min under a gradient program in mobile phase A (water: acetonitrile: formic acid 900:100:1) and mobile phase B (acetonitrile: water: formic acid 900:100:1). Please see Supplemental Experimental Procedures for specific separation parameters and experimental conditions.

### Data Processing and Statistical Data Analysis

Raw data processing is performed according to the instructions provided by the instrument manufacturer with some minor modifications (see Supplemental Experimental Procedures). Representative MS/MS spectra were exported in abf format for MS-DIAL, and compound identification was performed against MS/MS libraries. Single-factor variable analysis of material abundance data was analyzed by R-language T test, and PLS-DA was analyzed by R-language software package MetaboAnalystR. Hypergeometric distribution test was used for KEGG enrichment analysis, and “BH” method was used for false positive correction. *P* < 0.05 after correction was used as the screening threshold of significant enrichment pathway.

## Results

### Mtb Infection Reduces Gut Microbiota Diversity and Taxonomic Compositions in ATB Patients

**Fig. 1:**
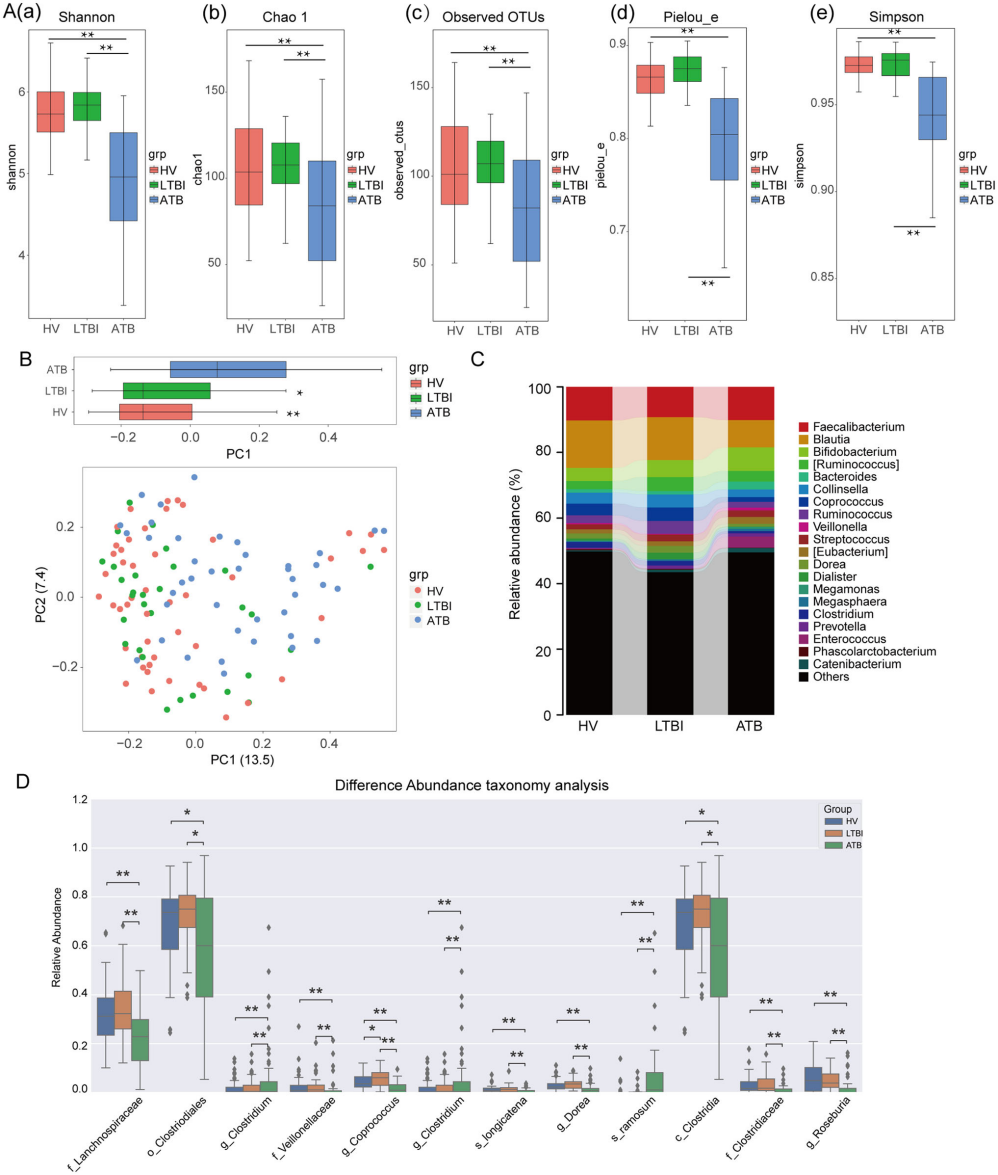
Mtb infection induces minor changes in the intestinal microbiota **A** Based on 16S rRNA sequencing data, *α* overall diversity analysis performed for gut sample in HV, LTBI and ATB groups in this study. Error bars indicate minimum and maximum values. Statistical significance was calculated among the different groups using Kruskal–Wallis test. *p < 0.05, **p < 0.01.**a** Shannon indices; **b** Chao 1 indices; **c** Observed OTUs; **d** Pielou_e indices; **e** Simpson indices; **B** PCA analysis is based on OTUs from the sequences of the V3–V4 regions of the 16S rRNA in 120 stool samples. Significant differences across groups are established at the first principal component (PC1) values, and shown in the box plots above. *P value < 0.05, Wilcoxon rank sum test. **C** Genus taxonomic distribution of the intestinal microbiota from subjects in above three groups. **D** Relative abundances of the most common organizational taxonomic units identified in fecal samples from HV, LTBI and ATB subjects. OTUs were compared by the Holm-Sidak method t-test for all OTUs, with average relative abundances of greater than 1% across all samples, and significant differences are demonstrated by asterisks (*P < 0.05; **P < 0.01; ***P < 0.001).

To assess the effect of Mtb infection on the gut microbiota in ATB patients, we analyzed the diversity and composition of gut microbiota using 16S rRNA gene sequencing in 120 fecal samples (V3–V4 region) from active tuberculosis patients without HRZE treatment (ATB, n = 41), volunteers with latent Mtb infection (LTBI, n = 30) and healthy volunteers (HV, n = 49). Detailed characteristics of recruited participants are shown in Table 1 and the research plan is shown in Fig. S1. Alpha diversity were estimated using Shannon, Chao 1, Observed OTUs, Pielou_e and Simpson indices for fecal sample collection in the ATB patients, LTBI and HV volunteers. Compared with LTBI or HV volunteers, the overall diversity of microbiota in the ATB patients was significantly decreased (Fig. 1A). Similarly, ATB patients could be demarcated from LTBI or HV volunteers using principal co-ordinates (PCoA) analysis based on Bray–Curtis metrics (Fig. 1B), suggesting Mtb infection could induce the changes of the gut microbiota. Permanova analysis also identified Mtb infection serves as the main driving force to cause variance among ATB patients, LTBI and HV volunteers (*P* < 0.001). However, the pattern of gut microbiota of LTBI volunteers was relatively close to that of HV volunteers.

We subsequently investigated the composition of gut microbiota to identify the different bacterial taxa among the ATB patients, LTBI and HV volunteers. We found the trends of differential relative abundance mainly in the genera *Blautia*, *Bacteroides*, *Coprococcus*, *Enterococcus* as well as certain members of the phylum Firmicutes among the ATB patients, LTBI and HV volunteers (Fig. 1C). The trends in differential abundance of other bacterial taxa were also shown in Fig. S2. To further determine which taxa were greatly influenced by Mtb infection, the Kruskal–Wallis H test was used to analyze 16S rRNA sequencing data to investigate remarkable changes at the genus level. ATB patients have a relative enrichment of genera *Clostridium* (family *Erysipelotrichaceae*) and *Clostridium ramosum* compared to LTBI or HV volunteers. Conversely, Mtb infection also caused a marked decrease in the relative abundance of family *Lachnospiraceae*, *Veillonellaceae*, *Clostridiaceae* and genera *Coprococcus*, *Dorea* and *Roseburia*, all belonging to family *Lachnospiraceae* of order Clostridiales. (Fig. 1D).

### Reduced Gut Microbiota Diversity is Restored in ATB Patients During Anti-TB Chemotherapy

Recently, some studies have shown that anti-TB chemotherapy can change the structure of gut microbiota [10, 11, 13]. However, most of these studies focused on the microbiota changes between healthy individuals and TB patients, little attention was paid to the changes of microbiota during anti-TB chemotherapy. Therefore, we examined the impact of anti-TB chemotherapy in gut microbiota of ATB patients in a three-way comparison of the active TB patients without HRZE treatment (ATB), TB with 2-month HRZE (T2) and TB with 6-month antibiotics (2-month HRZE followed with 4-month HR, T6) using the same methodology described above. Unexpectedly, we found that anti-TB chemotherapy for 6 months (T6) could cause a significant increase of the overall bacterial diversity in the gut microbiota, compared to ATB using Pielou’s evenness index and Simpson index (Fig. S3A). Then, we used Bray–Curtis PCoA analysis to compare the gut microbiota changes among ATB, T2 and T6 (Fig. S3B). The results showed a significantly separated clustering of samples in T6 from those of ATB but not from those of T2 suggesting a temporal change in community structure (*P* = 0.022).

In addition, we observed a dramatic alteration in the bacterial composition of gut microbiota at the genus level in T6 group, while no significant changes were observed in T2 group (Fig. S3C). We next to investigate which taxa were affected by anti-TB chemotherapy and a successive increase of family *Veillonellaceae*, family *Bacteroidaceae*, genera *Veillonella* (family *Veillonellaceae*), *Dialister* (family *Veillonellaceae*) and *Bacteroides* (family *Bacteroidaceae*) were found in the gut microbiota from TB patients at T2 and T6. Rather, genus *Clostridium* (family *Erysipelotrichaceae*) and its species *Clostridium ramosum* showed a successive decrease (Fig. S3D). Taken together, these results indicated that anti-TB chemotherapy using HRZE treatment at the 6th month had a significant effect on the composition of gut microbiota.

### Dynamic Regulation of Fecal Metabolome by Mtb in ATB Patients During Anti-TB Chemotherapy

The fecal metabolome can be used as a functional readout of the gut microbiome. Therefore, we further evaluate the relationship between fecal metabolome and gut microbiome in ATB patients during anti-TB chemotherapy. Herein, we performed non-targeted metabolomics profiling of fecal samples from ATB, T2, T6, and HV groups. A series of multivariate pattern recognition analyses including PCA and PLS-DA was carried out between the TB patients and HV volunteers. We firstly used PCA to reduce the number of variables in the metabolomics multivariate dataset (Fig. S4A) and then employed the PLS-DA analysis to obtain more reliable information about the differences between ATB and HV groups. The score map obtained by PLS-DA analysis (Fig. S4B) showed that the ATB patients and HV volunteers can be clearly distinguished. Subsequently, we identified 63 metabolites that differed in abundance between ATB patients and HV volunteers (Supplementary Table S2). Using above mentioned methods, we also further examined the effect of anti-TB chemotherapy on fecal metabolites. The score map of PLS-DA analysis showed that the ATB could be clearly distinguished from T6, but not from T2 (Fig. S4C and S4D). In anti-TB treatment, 53 fecal metabolites of ATB patients had significantly changed (Supplementary Table S3).

### Changes in Fecal Metabolites are Related to the Gut Microbiota Dysbiosis

**Fig. 2:**
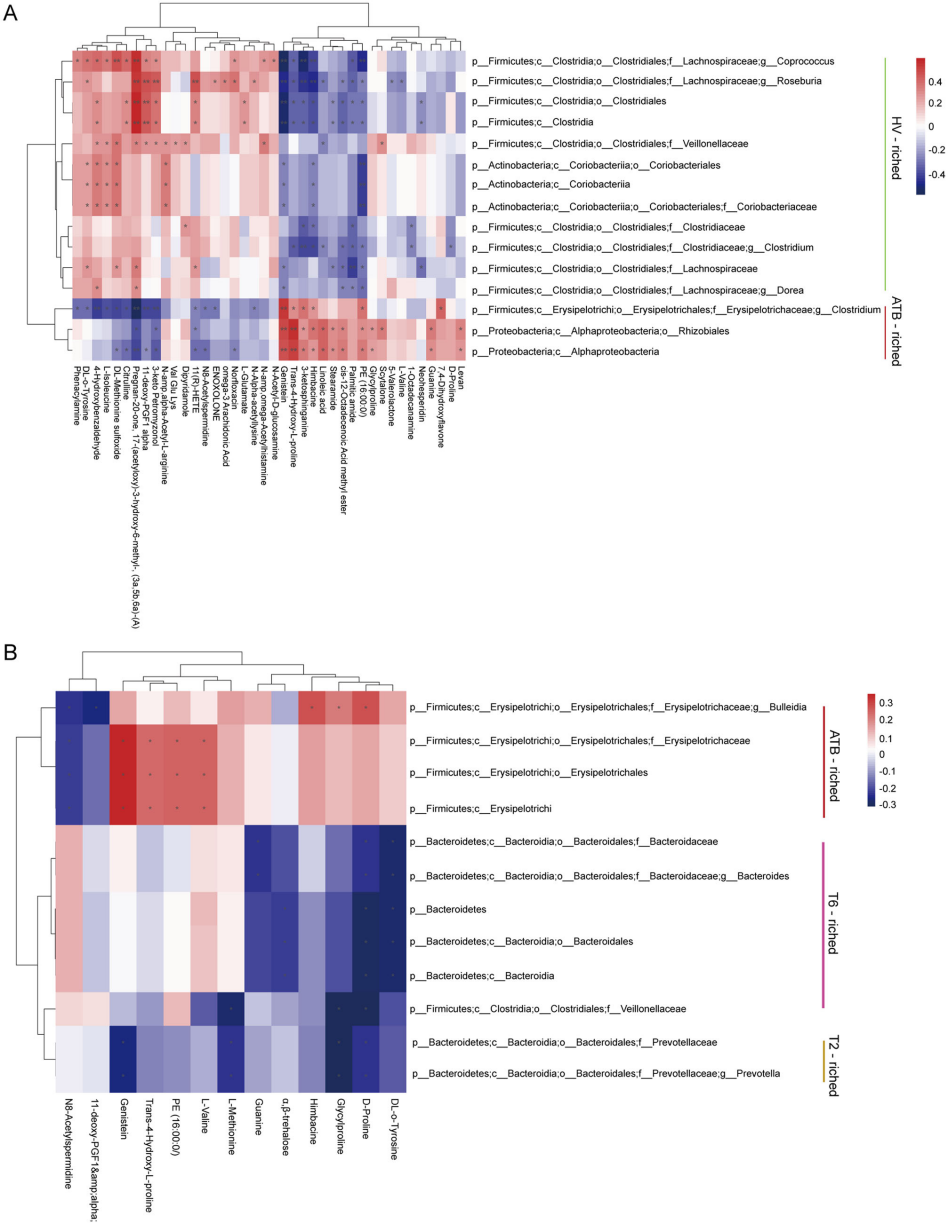
Aberrant metabolic patterns in stool samples caused by anti-TB therapy** A** Correlations between differential fecal metabolic patterns and bacteria caused by Mtb infected. Spearman’s correlation coefficients between the level of 63 reliable and markedly different fecal metabolic patterns and the abundance of the differentially enriched bacteria were calculated. Blue, negative correlation; Red, positive correlation, *q < 0.05. **B** Correlations between differential fecal metabolic patterns and bacteria caused by anti-TB therapy. Spearman’s correlation coefficients between the level of 53 reliable and markedly different fecal metabolic patterns and th abundance of the differentially enriched bacteria were calculated. Blue, negative correlation; Red, positive correlation, *q < 0.05.

To further investigate whether the changed abundance of fecal metabolites correlated with the altered gut microbiota, we used Spearman’s correlation analysis to find possible correlations between the altered gut microbiota and metabolites that differed in abundance between ATB patients and HV volunteers (Fig. 2A, Fig. S5 and Table S2). We found that some fecal metabolites such as phenacylamine, DL-*o*-Tyrosine, DL-Methionine sulfoxide, 4-Hydroxybenzaldehyde, L-Isoleucine, and 11(R)-HETE which decreased in ATB patients were negatively correlated with ATB patients enriched bacteria *Clostridium* (family *Erysipelotrichaceae*), while the increasing metabolites, such as *Trans*-4-Hydroxy-L-proline, Himbacine, PE (16:0/0:0), 7,4′-Dihydroxyflavone and Genistein, were positively correlated with *Clostridium*. In addition, bacteria enriched in HV controls, belonging to family *Lachnospiraceae*, such as *Coprococcus* and *Roseburia* were also negatively correlated with genistein, *Trans*-4-Hydroxy-L-proline, Himbacine and PE (16:0/0:0), while positively correlated with 11(R)-HETE and Pregnan-20-one,17-(acetyloxy)-3-hydroxy-6-methyl-, (3a,5b,6a)-(A) (Fig. 2A).

Using above mentioned methods, we also further analyzed the relationship between fecal bacteria and metabolites during anti-TB chemotherapy. Using Spearman’s correlation analysis, our results showed that fecal metabolites such as Glycylproline, D-proline, DL-*o*-Tyrosine and Guanine which decreased in TB patients at T6 were negatively correlated with T6-enriched bacteria *Bacteroides* (Fig. 2B and Fig. S6). Moreover, fecal metabolites such as Genistein, L-methionine, Glycylproline, D-proline and DL-*o*-Tyrosine which reduced in TB patients at T2 were negatively correlated with bacteria enriched in T2-enriched bacteria such as *Prevotella* (Fig. 2B). Taken together, these results suggest that the altered abundance of fecal metabolites correlated with the altered gut microbiota, especially the change of gut *Clostridium* (family *Erysipelotrichaceae*), *Coprococcus*, *Roseburia*, *Bacteroides*, and *Prevotella* is closely related to the change of fecal metabolites caused by Mtb infection.

## Discussion

This study presents the effects of anti-TB chemotherapy using standard HRZE on human gut microbiota and fecal metabolites of ATB patients. In agreement with previous studies [5, 12, 13], we observed that a minor intestinal flora disorder in ATB patients was associated with Mtb infection, which are mainly reflected in the overall diversity and structure of gut microbiota. In gut microbiota of ATB patients, we observed a decreased ratio of Firmicutes to Bacteroidetes, which can affect the concentration of SCFAs to regulate energy metabolism, lipid metabolism, cholesterol metabolism and systemic inflammatory response. Moreover, there was a significant decrease in SCFA-producing bacteria, viz. *Coprococcus*, *Dialister, Dorea, Collinesella, Anaerostipes, Roseburia* and *Phascolarctobacterium* (Fig. 1) and Fig. S5 [18, 19], which may lead to a potential decrease of SCFAs that can further result in metabolic disorders to affect the tuberculosis infection and prognosis.

Oral antibiotics are known to result in a disorder of gut microbiota, and often associated with side effects [20]. Although most anti-TB agents (HRZE) belong to narrow-spectra, compared to the broad-spectrum antibacterial agents, understanding their effects on microbiota also has important implications for the treatment and reinfection of tuberculosis. Our data indicate that the overall diversity of intestinal microflora in HRZE-treated cases has undergone major changes during anti-TB chemotherapy (Fig. S3). The most noteworthiness is the depletion of *Clostridium* (family *Erysipelotrichaceae*) along with an increase of *Veillonella*, *Bacterioides* and *Dialister *(Fig. S3). The consequences of HRZE-induced taxonomic disruption in the gut microbiota are currently unknown, but some of these bacteria have been related to TB immunity. For example, Shen et al*.* have reported that the polysaccharide in *Bacterioides* can regulate host inflammatory responses in mice [21]. Farshad Nojoomi et al*.* have pointed that *Veillonella* contained some anti-inflammatory properties [22]. *Dialister* possess potent anti-inflammatory properties due to its ability to produce large amounts of SCFAs, such as propionate and lactate. Besides, *Clostridium* (family *Erysipelotrichaceae*) and its species *Clostridium ramosum* had an obvious increase in ATB and were significantly reduced after anti-TB treatment. *Clostridium ramosum* was reported to cause a downregulation of PPAR-γ expression in the intestine [23], suggesting that it may aggravate Mtb infection. Above all, these results indicate that the perturbation of the microbiota induced by HRZE may dramatically affect human immune responses, so that it can affect the efficacy of anti-TB chemotherapy.

The communication between the host and its microbiome occurs partly through the secretion of metabolites, which have a profound influence on the physiological function of the host. The immune system can continuously scan the intestinal microenvironment to obtain information about the metabolic status and colonization status of the microbiota. Recent studies have uncovered that microbial metabolites play an important role in regulating the immune system [24]. By altering the production of butyric acid and propionic acid, the intestinal microbiota leads to impaired immune function in TB patients [10]. Negatu DA et al. have revealed that the intestinal microbial metabolite indolepropionic acid targets tryptophan to interfere with the biosynthesis of Mtb [25]. Here, through metabolomic analysis, we found that Mtb infection can give rise to obvious changes in the relative abundance of 63 fecal metabolites and some metabolites have also been significantly changed during anti-TB chemotherapy.

In these metabolites, some altered lipids in ATB patients are also associated with the arachidonic acid metabolism, such as the 11(R)-HETE and omega-3 arachidonic acid, as well as 3-ketosphinganine, Linoleic acid, Stearamide, *cis*-12-Octadecenoic Acid methyl ester, palmitic acid and PE (16:0/0:0). 11(R)-HETE is an arachidonic acid metabolite produced by both COX-1 and COX-2 (cyclooxygenases), which is expressed in human and murine macrophages and induced by LPS, interleukin 1 (IL-1) and phorbol esters. The expression level of COX-2 can regulate the autophagy and bactericidal activities of macrophages infected by Mtb. COX-2-derived PGs (PGD, PGE, PGF and PGI) serve as anti-inflammatory and anti-fibrotic lipid mediator to fight inflammation [26]. In addition, omega-3 arachidonic acid has been shown to promote mycobacteria killing by activating NF-κB. Therefore, it is necessary to further study the role of arachidonic acid metabolism in host defense against Mtb.

It has been reported that in inflammatory response, the palmitic acid and its derivatives in the endoplasmic reticulum (ER), on the one hand, can increase the reactive oxygen species (ROS) generation, leading to cell death; on the other hand, they also drive the activation of NF-κB and NLRP3, facilitating the release of proinflammatory cytokine by monocytes/macrophages [27]. José Marcos Sanches et al*.* had also shown that certain potential lipid biomarkers in macrophages, such as palmitic acid and PE(16:0/0:0), are released after NLRP3 activation that can modulate the inflammatory responses in the damaged tissue [28].

Except the lipids, we also found that some amino acids, such as l-methionine, glycylproline, D-proline and DL-*o*-Tyrosine were decreased in ATB patients post anti-TB chemotherapy. D-amino acids are involved in the synthesis and cross-linking of bacterial peptidoglycan. Hochbaum et al*.* had reported that D-phenylalanine, D-proline and D-tyrosine can inhibit the biofilm formation in *Staphylococcus aureu* [29]. Proline-derived *trans*-4-Hydroxy-L-proline (Hyp) was proposed as diagnostic markers in urine of TB. It is also one of the principal components of collagen triplets. Pro-hyp-gly is the most common collagen triplet (10.5%), since these residues contribute greatly to the stability of the trihelix. The collagen structure and the enzymes responsible for collagen destruction are considered potential targets for adjuvant treatment of TB. The Th2 cells were reported to promote a fibrotic reaction characterized by collagen III expression and formation of Hyp [30].

O-tyrosine has been reported as a potential marker of oxidative stress in acute and chronic diseases associated with oxidative stress. Supplementation of para-tyrosine to compete with o-tyrosine may play a protective role in oxidative stress-related diseases [31]. In addition, Vrieling et al. found that plasma methionine, glycylproline in TB patients decreased levels at 2 months followed by a rise at 26 weeks post-treatment [32]. In our study, the similar effects were observed in the fecal methionine and glycylproline.

Interestingly, genistein was increased in ATB patients, while decreased during anti-TB treatment. Mezei et al*.* demonstrated that genistein and 7,4′-dihydroxyisoflavone (the isomer of 7,4′-dihydroxyflavone) significantly increased PPARγ-directed gene expression in RAW 264.7 cells which can regulate macrophage apoptosis during Mtb infection [33].

And, more remarkable, using Spearman’s correlation analysis, our results showed that the increased metabolites, such as Hyp and Genistein, were positively correlated with *Clostridium* (family *Erysipelotrichaceae*) in ATB patients, while negatively correlated with *Roseburia* and *Coprococcus* in HV controls*.* D-Proline and DL-*o*-Tyrosine were negatively correlated with T6-enriched bacteria *Bacteroides.* Genistein was negatively correlated with T2-enriched bacteria *Prevotella* while positively correlated with ATB-enriched bacteria *Clostridium.* In RAW264.7 cells, genistein had been reported to significantly attenuate the production of iNOS-derived NO and IL-6 induced by *Prevotella intermedia* LPS, and their mRNA expression were also decreased [34]. And the low efficiency of NO can inhibit the antimicrobial macrophages function. Hyp dehydratase (HypD) was found in Clostridiales and genera *Clostridium*, *Bacteroides* and *Enterococcus* in human stool samples, suggesting that it may be the cause of the anaerobic Hyp degradation. The HypD-encoding species in gut *Bacteroides* can catabolize the host-derived polysaccharides covalently linked to protein residues and large glycoprotein to access Hyp and other amino acids to provide amino acids to gut microbes [35]. However, the detailed relationship between these metabolites and bacteria is unknown. Future studies are needed to explore the causality and investigate the specific mechanisms, so that relevant intervention strategies can be proposed.

## Conclusion

TB patients treated with HRZE can disrupt both gut microbiota and metabolites. Some specific genera and their related metabolites are depleted or enriched after treatment. The genera *Veillonella* (Family *Veillonellacea*) and *Bacteroides* (Family *Bateroidaceae*) significantly increased in the gut microbiota during anti-TB chemotherapy. And the change of gut *Clostridium*, *Bacteroides* and *Prevotella* was closely related to the change of fecal metabolites such as *Trans*-4-Hydroxy-L-proline and Genistein caused by Mtb infection or anti-TB chemotherapy. These findings, to some extent, provide direct evidence to verify the hypothesis of intestinal dysbacteriosis in patients with anti-TB chemotherapy and to comprehensively understand the correlation between TB and intestinal microbiota. However, this preliminary clinical study result has some limitations, because the participants included in this study have similar backgrounds and characteristics, and each group has a small number of participants, so this study should be further expanded and improved to verify these results in a larger cohort study.

## Supplementary Information

Below is the link to the electronic supplementary material.

Supplementary file1 (DOCX 37 KB)

Supplementary file2 (XLSX 155 KB)

Supplementary file3 (XLS 524 KB)

## Data Availability

The datasets generated during and/or analyzed during the current study are available from the corresponding authors upon request.
