# Supplementary material for: Clostridium, Bacteroides and Prevotella associates with increased fecal metabolites Trans-4-Hydroxy-L-proline and Genistein in active pulmonary tuberculosis patients during anti-tuberculosis chemotherapy with isoniazid-rifampin-pyrazinamide-ethambutol (HRZE)
Source: Indian J Microbiol. 2022 Mar 24;62(3):374–83. doi: 10.1007/s12088-022-01003-2 (PMC9375812; doi:10.1007/s12088-022-01003-2)
Supplement: Supplementary file 1 — Supplementary file1 (DOCX 37 KB) [file 12088_2022_1003_MOESM1_ESM.docx]

**Supplemental Materials**

**For**

***Clostridium*, *Bacteroides* and *Prevotella* associates with increased fecal metabolites Trans-4-Hydroxy-L-proline and Genistein in active pulmonary tuberculosis patients** **during anti-tuberculosis chemotherapy with isoniazid-rifampin-pyrazinamide-ethambutol** **(HRZE)**

**Supplemental Experimental Procedures**

**Ethics Approval and consent to participate**

All the participants in the investigation were enrolled through the Shenzhen Center for Chronic Disease Control and gave their written informed consent. This study has been approved by the Ethics Committee in Shenzhen Center for Chronic Disease Control. The research was carried out in accordance with the regulations and best practices of Shenzhen Center for Chronic Disease Control and Center for Tuberculosis Control of Guangdong Province.

**Subjects in this study**

The volunteers in this study were enrolled and subjected to analysis using IFN-γ release assay (QuantiFERON-TB Gold In-Tube (QFT), Qiagen, CA, USA) along with clinical, microbiological, and radiographical examinations. The criteria for enrollment were as follows: (1) patients with active TB (ATB group in this study) showed clinical and radiographical features of tuberculosis and were confirmed by sputum smear or culture. Moreover, they did not receive anti-TB treatment before sample selection. (2) All the controls (IGRA- and IGRA+) were with TB-resembling coughing symptoms and normal X-ray but being culture-negative and without other clinical TB symptoms and TB contact history. Exclusion criteria included a history of antibiotic or probiotic treatment more than 1 week within the previous 8 weeks. We recruited five groups of individuals using a cross-sectional research study design. Characteristics of the study cases are given in Table 1. 49 without Mtb infection (IGRA-), 30 latently infected by Mtb (LTBI), 41 active TB, 28 patients with 2-month HRZE treatment and 20 patients with 2HRZE/4HR treatment were enrolled to assess the effect of Mtb on the gut microbiota as well as the impact of anti-TB treatment on gut microbiota. All subjects are HIV negative and without diabetes history.

**DNA isolation and 16S rRNA genes sequencing library preparation**

Fecal genomic DNA samples were extracted using the Fast DNA SPIN extraction kits (MP Biomedicals, Santa Ana, CA, USA), following the manufacturer’s instructions, and stored at −20 °C for further analysis. PCR ampliﬁcation of the bacterial 16S rRNA genes was performed using the forward primer 338F (5’-ACTCCTACGGGAGGCAGCA-3’) and the reverse primer 806R (5’-GGACTACHVGGGTWTCTAAT-3’) for V3-V4 region of gut microbiota. Sample-specific 7-bp barcodes were incorporated into the primers for multiplex sequencing. Thermal cycling consisted of initial denaturation at 98 °C for 2 min, followed by 25 cycles of 15 s at 98 °C, annealing at 55 °C for 30 s, and extension at 72 °C for 30 s, with a final extension of 5 min at 72 °C. PCR amplicons were puriﬁed with Agencourt AMPure Beads (Beckman Coulter, Indianapolis, IN) and quantiﬁed using the PicoGreen dsDNA Assay Kit (Invitrogen, Carlsbad, CA, USA). After the individual quantification step, amplicons were pooled in equal amounts, and pair-end 2X 300 bp sequencing was performed using the Illlumina MiSeq platform with MiSeq Reagent Kit v3 at Shanghai Personal Biotechnology Co., Ltd (Shanghai, China).

**DNA sequencing and data processing**

Bioinformatic analysis of the bacterial 16S rRNA amplicon data was conducted using a custom Quantitative Insights Into Microbial Ecology (QIIME2) software pipeline (QIIME2 V.2019.10). Sequence quality control and filtering were conducted by FastQC v.0.11.2 and Trimmomatic v.0.32, followed by DADA2 software, wrapped in QIIME2, was used to filter the sequencing reads and construct feature table (amplicon sequence variants (ASVs) and its absolute abundance). The taxonomy of each 16S rRNA gene sequence was assigned by q2-feature-classifier (QIIME2 V.2019.10) based on the feature table. Pre-trained Naive Bayes taxonomy classifier gg-13-8-99-515-806-nb-classifier was used in the classification.

**Bioinformatics and Statistical Analysis**

Sequence data analyses were mainly performed using QIIME2, Python (v3.7) and R packages (v3.6.3). Alpha diversity was performed via q2-diversity alpha-phylogenetic based on the feature table (the metric observed OTUs was calculated by ASVs, but in order to align the definition with Qiime2, we keep the name of observed OTUs). Beta diversity analysis was performed to investigate the structural variation of microbial communities across samples using UniFrac distance metrics [1] and visualized via Principal Co-ordinates Analysis (PCoA) [2]. Differences in the Unifrac distances for pairwise comparisons among groups were determined using Student’s t-test and the Monte Carlo permutation test with 1000 permutations, and using Tukey’s multiple-comparisons test when appropriate [2]. The significance of differentiation of microbiota structure among groups was assessed by PERMANOVA (Permutational multivariate analysis of variance) [3] and ANOSIM (Analysis of similarities) using R package “vegan” [4, 5].

Taxa abundances at the phylum, class, order, family, genus and species levels were statistically compared among samples or groups by Kruskal-Wallis test or LEfSe (Linear discriminant analysis effect size) which was performed to detect differentially abundant taxa across groups using the default parameters [6]. Correlation analysis was carried out by using R language to analyze the relative abundance of metabolites detected and the relative abundance of bacteria. The correlation analysis method was Spearman. The metabolic data were selected from 64 metabolites with a high degree of substance identification and a detection rate of more than 50% among samples. The 16s data were selected from the relative abundance data of genus level and the abundance data of bacteria with significant difference in LefSE analysis. The results were shown by the correlation heat map, in which the color represented the correlation coefficient, * indicated that the correlation P value was less than 0.05, and ** indicated that the correlation P value was less than 0.001. All other plots were made by Prism 7.

**Metabolite extraction for LC-MS**

Fecal samples were reconstituted with PBS and centrifuged at 3,000 rpm for 30 min to obtain the supernatant which was filtered twice using 0.22 µm filters (Millipore, Billerica, MA, USA). Supernatant was reconstituted by dissolving in 1 mL solvent mixture containing methanol/acetonitrile (1∶1). The samples were vortexed for 30 s and -20 °C for 2 h. Then centrifuged at 13,000 g for 15 min at 4 °C and transferred to LC vial for LC–MS analysis. Quality control (QC) samples were prepared by mixing each aliquot with a pooled sample and analyzing them in parallel using the same method. The QCs were injected at regular intervals (every 8 samples) throughout the analytical run to provide a set of data from which repeatability could be assessed.

**Identification of metabolites by LC-MS**

The separation was performed by Ekspert UltraLC (110, AB Sciex) and equipped with ACQUITY UPLC HSS T3 (1.8 μm 2.1×100 mm, Waters) column at a flow rate of 0.3 mL/min under a gradient program in mobile phase A (water: acetonitrile: formic acid 900:100:1) and mobile phase B (acetonitrile: water: formic acid 900:100:1). The gradient program was applied as follows: t=4 min, 0% B; t=6 min, 25% B; t=25min, 100% B; t=29.1 min, 100% B; t=31 min, 0% B; t=33 min, 0% B. The stop time was 40 min. For MS analysis, data were acquired by AB 5600 + Triple TOF mass spectrometer (SCIEX, Redwood City, CA, USA) operating in the positive ion mode. The capillary voltage was set at + 5500 V (positive mode). Other source conditions were kept constant in all the experiments as follows: the pressure of nebulizer gas (nitrogen) was 40 pa. The sheath gas was maintained at a temperature of 550 °C. The scan range was adjusted to 100–1200 m/z.

**Data processing and statistical data analysis**

The raw MS files (WIFF format file) were converted to ABF (analysis base file format) using the freely available Reifycs file converter (http://www.reifycs.com/AbfConverter/). Peak picking and alignment were performed using MS-DIAL version 3.70 and the parameters were set as follows: Alignment: MS1 tolerance, 0.015 Da; Retention time tolerance, 0.3min; Identification: Accurate mass tolerance (MS1), 0.01Da; Accurate mass tolerance(MS2), 0.05Da. Representative MS/MS spectra were exported in abf format for MS-DIAL, and compound identification was performed against MS/MS libraries including METLIN [7], MassBank [8] and MONA [9]. Single-factor variable analysis of material abundance data was analyzed by R-language T test, and PLS-DA was analyzed by R-language software package MetaboAnalystR. Hypergeometric distribution test was used for KEGG enrichment analysis, and "BH" method was used for false positive correction. P < 0.05 after correction was used as the screening threshold of significant enrichment pathway.

**Supplementary Table S1.** **Characteristics of the participants enrolled for metabolome analysis in this study.**

Data are divided into the study groups described in the text. The number of subjects, average age, gender distribution and time on HRZE treatment are shown. Healthy volunteers are IGRA−. Active tuberculosis patients were finally diagnosed by positive test of Mtb in sputum specimens.

| Group^a^ | No. of subjects | Age, mean ± SD, year | Male, n(%) | Anti-TB duration | No. of reads | No. of OTUs | Diagnosis |
| --- | --- | --- | --- | --- | --- | --- | --- |
| HV | 28 | 38.5±11.8 | 8(29.6) | N/A | 105839.9±  13623.5 | 93.0±21.5 | Mtb-/IGRA- |
| ATB | 33 | 35.6±12.0 | 22(66.7) | N/A | 119036.1±  43303.5  1 | 78.8±32.5 | Mtb+ |
| T2 | 28 | 34.2±10.7 | 22(75) | 2 months | 108212.1±  14328.4 | 68.6±28.4 | - |
| T6 | 18 | 39.4±13.3 | 11(61.1) | 6 months | 94627.8±  20775.0 | 64.5±22.4 | - |

^a^HV: Mtb uninfected; LTBI: individuals with latent TB infection; ATB: Active TB; T2: TB patients with anti-TB therapy (HRZE) for 2 months; T6: TB patients with anti-TB therapy (HRZE) for 6 months.

**Supplemental Table S2.** **Differential metabolites of stool samples in ATB subjects compared to those in HV subjects identified by LC-MS metabonomics.**

**Supplemental Table S3. Differential metabolites of stool samples in T2 and T6 subjects respectively compared to those in ATB subjects identified by LC-MS metabonomics.**

**Supplementary Figures**

**Supplemental Figure S1.** **Outline of experimental scheme for exploration of alterations in the gut microbiota and metabolites induced by Mtb infection and/or anti-TB therapy.**

The healthy controls contain two groups, viz. healthy volunteers (HV) and volunteers with latent Mtb infection (LTBI). TB patients consisted of three groups. ATB, active TB patients without HRZE treatment; T2, patients treated by HRZE for 2 months; T6, patients with anti-TB therapy for 6 months. For the purpose of further detecting the effect caused by HRZE drugs, TB patients were employed to track the change of their intestinal microbiota with standard anti-TB treatment for two months and six months, respectively.

**Supplemental Figure S2.** **The five taxonomic distribution of the intestinal microbiota from subjects in HV, LTBI, ATB, T2 and T6 groups.**

**Supplemental Figure S3. HRZE treatment changes the overall community structure and composition of the gut microbiota.**

**(A)** α diversity was calculated by the Simpson index and Pielou's evenness index, which all revealed significant differences among active TB without HRZE treatment (ATB), TB treated for 2 months (T2) and TB treated for 6 months (T6) groups. *P<0.05, **P<0.01, ***P<0.001. **(B)** PCA analysis is based on OTUs from the sequences of the V3-V4 regions of the 16S rRNA in 89 stool samples. Significant differences across groups are established at the first principal component (PC1) values, and shown in the box plots above. *P value <0.05, Wilcoxon rank sum test. **(C)** Genus taxonomic distribution of the intestinal microbiota from subjects in ATB, T2 and T6 groups. **(D)** Relative abundances of the most common organizational taxonomic units identified in stool samples from ATB, T2 and T6 subjects. OTUs were compared by the Holm-Sidak method t-test for all OTUs, with average relative abundances of greater than 1% across all samples, and significant differences are demonstrated by asterisks (*P<0.05; **P<0.01; ***P<0.001). Some genus designations appear more than once because multiple OTUs have the same consensus taxonomy.

**Supplemental Figure S4. Alterations in the gut metabolites induced by Mtb infection or anti-TB therapy.**

**(A)** PCA score plots based on the metabolic profiles in stool samples from HV, ATB, T2 and T6 group. **(B)** Score scatter plots of PLS-DA comparing the metabolic differences identify the separation between HV and ATB. **(C)** Score scatter plots of PLS-DA comparing the metabolic differences identify the separation between ATB and T2. (D) Score scatter plots of PLS-DA comparing the metabolic differences identify the separation between ATB and T6.

**Supplementary Figure S5. LEfSe comparisons showing the differentially abundant genera in the gut microbiota between the HV and ATB groups.** The bacteria significantly enriched in HV and ATB groups are shown with blue and red bars respectively. Data are filtered for P < 0.05 and linear discriminant analysis (LDA) score > 2.

**Supplementary Figure S6. LEfSe comparisons showing the differentially abundant genera in the gut microbiota between the ATB, T2 and T6 groups.** The bacteria significantly enriched in ATB, T2 and T6 groups are shown with red, blue and green bars respectively. Data are filtered for P < 0.05 and linear discriminant analysis (LDA) score > 2.

**Reference**

[1] Lozupone C A, M Hamady, S T Kelley, et al., (2007) Quantitative and qualitative beta diversity measures lead to different insights into factors that structure microbial communities. Appl Environ Microbiol 1576-85 <http://dx.doi.org/10.1128/AEM.01996-06>

[2] Ramette A, (2007) Multivariate analyses in microbial ecology. FEMS Microbiol Ecol 142-60 <http://dx.doi.org/10.1111/j.1574-6941.2007.00375.x>

[3] McArdle B H A, M.J. , (2001) Fitting multivariate models to community data: a comment on distancebased redundancy analysis. Ecology 290-297

[4] Clarke K R, (1993) Non-parametric multivariate analyses of changes in community structure. Australian Journal of Ecology 117-143

[5] Warton D I, S T Wright, Y Wang, (2012) Distance-based multivariate analyses confound location and dispersion effects. Methods in Ecology and Evolution 89-101 <http://dx.doi.org/10.1111/j.2041-210X.2011.00127.x>

[6] Segata N, J Izard, L Waldron, et al., (2011) Metagenomic biomarker discovery and explanation. Genome Biol R60 <http://dx.doi.org/10.1186/gb-2011-12-6-r60>

[7] Guijas C, J R Montenegro-Burke, X Domingo-Almenara, et al., (2018) METLIN: A Technology Platform for Identifying Knowns and Unknowns. Anal Chem 3156-3164 <http://dx.doi.org/10.1021/acs.analchem.7b04424>

[8] Horai H, M Arita, S Kanaya, et al., (2010) MassBank: a public repository for sharing mass spectral data for life sciences. J Mass Spectrom 703-14 <http://dx.doi.org/10.1002/jms.1777>

[9] Hilbig M, M Rarey, (2015) MONA 2: A Light Cheminformatics Platform for Interactive Compound Library Processing. J Chem Inf Model 2071-8 <http://dx.doi.org/10.1021/acs.jcim.5b00292>
